# Prolonged loss of intercostal muscle mass and its predictors in COVID-19 patients: A retrospective study from tertiary hospital

**DOI:** 10.1097/MD.0000000000038284

**Published:** 2024-05-31

**Authors:** Byeong Ju Koo, Ho Cheol Choi, Hye Young Choi, Hwa Seon Shin, Jung Ho Won, Seok Jin Hong, Won Jeong Yang, Jae Kyeong Ahn, Mi Jung Park

**Affiliations:** a Department of Medicine, Gyeongsang National University College of Medicine, Jinju, Gyeongsangnam-do, South Korea; b Department of Radiology, Gyeongsang National University School of Medicine, Gyeongsang National University Hospital, Jinju, Gyeongsangnam-do, South Korea.

**Keywords:** COVID-19, sarcopenia, thoracic wall

## Abstract

Sarcopenia is a contributing factor in the development of long-COVID syndrome. We aimed to investigate how intercostal muscle mass changes over 3 months compared to other chest wall muscles following COVID-19 infection, along with identifying factors contributing to intercostal muscle loss during follow-up. We retrospectively studied 110 COVID-19 patients, analyzing muscle masses in the intercostal, pectoralis, and thoracic 12th vertebra level (T12) on initial and follow-up CT scans. Muscle mass was quantitatively assessed using density histogram analysis. We calculated the muscle difference ratio (MDR) as the following formula: (initial muscle mass - follow-up muscle mass)/initial muscle mass. Patients were categorized into 2 groups: <3 months follow-up (n = 53) and ≥ 3 months follow-up (n = 57). We employed stepwise logistic regression, using intercostal MDR ≥ 25% in follow-up as an independent variable and age < 65 years, ventilator use, steroid use, follow-up > 3 months, hospital stay > 13 days, body mass index < 18.5 kg/m², and female gender as dependent variables. The loss of intercostal muscle was the most severe among the 3 chest wall muscles in the CT follow-up. Intercostal MDR was significantly higher in the ≥ 3 months follow-up group compared to the < 3 months group (32.5 ± 23.6% vs 19.0 ± 21.1%, *P* = .002). There were no significant differences in pectoralis MDR or T12 MDR between the 2 groups. Stepwise logistic regression identified steroid use (3.494 (1.419–8.604), *P* = .007) and a follow-up period > 3 months [3.006 (1.339–6.748), *P* = .008] as predictors of intercostal MDR ≥ 25%. The intercostal muscle wasting was profound compared to that in the pectoralis and T12 skeletal muscles in a follow-up CT scan, and the intercostal muscle wasting was further aggravated after 3 months of COVID-19 infection. The use of steroids and a follow-up period exceeding 3 months were significant predictors for ≥ 25% of intercostal muscle wasting in follow-up.

## 1. Introduction

COVID-19 was declared a pandemic by the World Health Organization in 2020, leading to a significant number of hospitalizations due to COVID-19 pneumonia. Many individuals experience prolonged fatigue, muscle weakness, sleep disturbances, and mood disorders after recovering from COVID-19 pneumonia and subsequently encounter difficulties in having ordinary lives.^[1–6]^ If various systemic symptoms and signs persist more than 3 months after SARS-CoV-2 infection and no other disease has been diagnosed, the World Health Organization defines this condition as post-COVID-19.

The acute inflammation caused by conditions such as SARS-CoV-2 can lead to sarcopenia. Epithelial and endothelial cell damage, along with vascular leakage, can initiate a cytokine cascade, resulting in a massive inflammatory reaction.^[7,8]^ Proinflammatory cytokines, such as C-reactive protein, interleukin-6, and tumor necrosis factor-alpha, can increase and contribute to metabolic degradation and proteolysis.^[7,9]^ Previous studies have reported that patients with COVID-19 can lose 5 to 10% of their body weight during the acute infectious phase that lasts <2 weeks.^[7,10]^ Factors such as malnutrition, immobilization due to lockdown, and prolonged hospitalization further accelerate the development of sarcopenia and muscle wasting.^[8]^

In patients with COVID-19, chest CT is frequently utilized to assess the severity of pneumonia.^[11]^ In addition, CT offers the advantage of evaluating chest wall masses without additional radiation exposure. Previous studies have revealed the prognostic significance of chest wall muscle in patients with COVID-19 pneumonia, and the loss of chest wall mass in initial CT images is an independent prognostic factor for longer hospital stays, the need for mechanical ventilation, and overall survival.^[1–6,12]^ In previous studies, the chest wall mass was usually measured in pectoralis muscle, paraspinal muscle or whole skeletal muscle at the thoracic 12th vertebra level (T12).^[1–6,12]^

Even though the intercostal muscle plays a crucial role in respiration, the role of intercostal muscle mass in patients with pneumonia has been poorly demonstrated in previous studies. A previous study demonstrated that the loss of intercostal muscle mass on CT is associated with the severity of chronic obstructive pulmonary disease and the extent of emphysema.^[13]^ We hypothesized that intercostal muscle atrophy might occur not only in chronic illnesses but also in acute infections like pneumonia. We aimed to examine whether the decrease in intercostal muscle mass differed from the decrease in pectoralis muscle mass and T12 skeletal muscle mass. Furthermore, most studies have assessed chest wall masses in initial CT scans, and there have been no reports on the serial changes in chest wall masses. Our study is the first to measure the degree of sarcopenia in the different parts of the chest wall using serial CT images.

The purpose of our study was to examine how intercostal muscle mass changes 3 months after COVID-19 infection, unlike other chest wall muscles, such as pectoralis muscle, and whole skeletal muscle mass at T12.

## 2. Materials and methods

### 2.1. Study population

Our study included patients with COVID-19 pneumonia who were admitted to the hospital from January 2020 to March 2023. We retrospectively collected medical data from a single tertiary hospital that operates a severe emergency care center for COVID-19. The demographic data, such as age, height, weight, DM, hypertension, cardiovascular disease, end-stage renal disease, malignancy, usage of mechanical ventilation, steroid treatment, dates of polymerase chain reaction (PCR) test for COVID-19, dates of initial and follow-CT, and duration of hospitalization for all patients, were collected from electronic medical records for all patients. All patients were confirmed through PCR tests for COVID-19. Chest CT scans were conducted at admission and during follow-up, and pneumonia was found in the initial CT. We included patients with positive PCR test results within 7 days after chest CT and <20 days before chest CT. Patients were excluded if they had no follow-up CT (n = 175), missing clinical data (n = 23), a long duration between the positive PCR test results and chest CT (n = 18), a large amount of pleural effusion or subcutaneous edema (n = 3), or poor image quality of the chest CT (n = 6). Post-COVID-19 syndrome refers to systemic disease, such as muscle weakness, persisting for more than 3 months after a COVID-19 infection. Therefore, the patient group was divided into 2 groups based on a 3 months follow-up as cutoff point. Our study was approved by the institutional review board of our hospital (GNUH 2023-09-018), and the requirement for written informed consent was waived by the institutional review board.

### 2.2. Chest CT scan

Chest CT scans were performed at admission and follow-up using a third-generation dual-source CT scanner (Somatom Force; Siemens Healthcare, Forchheim, Germany). The following parameters were applied for this study: scan collimation of 0.6 mm × 192 slices, 0.75-mm slice thickness with 0.5 mm increments, tube voltage of 100 kVp, tube current of 100 mAs, gantry rotation time of 0.5 seconds, and pitch factor of 1.0. Contrast media were not administered during this study. All patients were scanned craniocaudally from the apex of the lung to the level of the diaphragm throughout maximal inhalation. The raw data were reconstructed employing a soft-tissue kernel (Br40) with a slice thickness of 2 mm and a slice interval of 2 mm.

### 2.3. Skeletal muscle assessment

**Figure 1.**
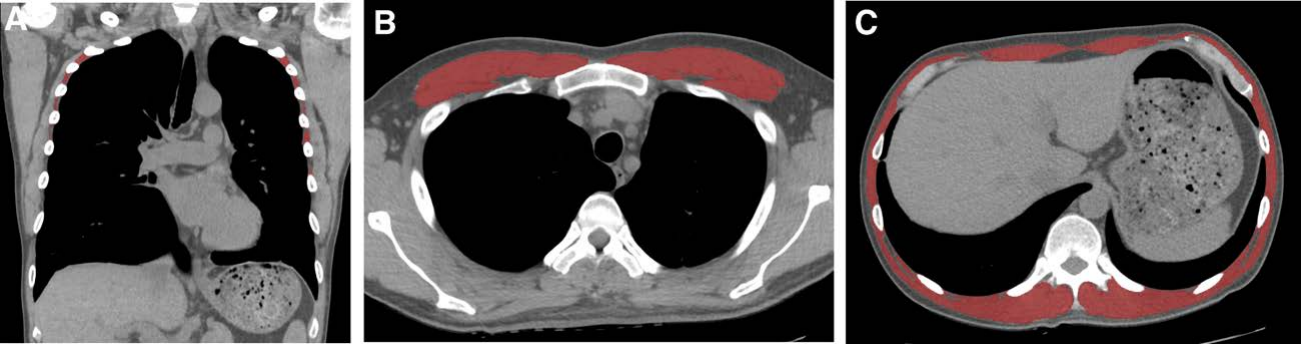
Muscle area measurement using non-contrast chest CT in patients with COVID-19. The region of interest (ROI) was manually delineated along the outermost border of the muscle, and muscle areas from −29 to 100 HU were displayed in pink and subsequently quantified using CT histogram analysis. (A) On a coronal image, the cross-sectional areas of both 3^rd^ to 8^th^ intercostal muscles were measured at the midline level. (B) On an axial image, the cross-sectional areas of both pectoralis major and minor muscles were measured at the 4^th^ level of the thoracic vertebra. (C) On an axial image, the cross-sectional areas of all the muscles were measured at the 12^th^ level of the thoracic vertebra. HU = Hounsfield units.


Two radiologists measured the skeletal muscle area in 3 different locations, including the intercostal, pectoralis and T12 skeletal muscle. The measurement of each muscle mass was independently performed by 2 radiologists: one with 13 years of experience as a chest radiologist, and the other with 4 years of experience. We conducted the measurements without access of the patients’ information. First, axial or coronal images were uploaded in 3D Slicer software platform (SLICER 3D 4.1.1, http://www.slicer.org). The mass of 3 skeletal muscles was analyzed using the ‘chest imaging platform’ extension (https://cip.bwh.harvard.edu/slicercip-workstation/) of the image computing. Second, the region of interest was manually drawn along the outermost border of the muscle using freehand drawing. Intercostal muscle mass was measured in a coronal plane image at the midline. We excluded the 1^st^, 2^nd^, and 9^th^ to 12^th^ intercostal muscles because it was difficult to distinguish their boundaries from the surrounding chest wall and diaphragm.^[13]^ Therefore, we measured the area of both the 3^rd^ and 8^th^ intercostal muscles. Pectoralis muscle mass measurements were obtained by measuring both pectoralis major and minor muscles at the 4^th^ thoracic vertebra on axial images. The axial image closest to the inferior border of the 12^th^ thoracic vertebra was chosen to measure the muscle mass, including the external and internal intercostal muscles, latissimus dorsi, rectus abdominis, external and internal oblique, and erector spinae^[4]^ (Fig. 1). Third, these muscle areas were automatically quantified with values ranging from −29 to 100 Hounsfield units using CT histogram analysis. Fourth, each muscle mass index in initial CT was defined as the muscle mass area divided by the square of the height. Fifth, we obtained the degree of muscle loss in the initial and follow-up CT with the following equation and defined it as the muscle difference ratio (MDR). The intercostal MDR, pectoralis MDR and T12 MDR were calculated from the intercostal muscle area, pectoralis muscle area and T12 muscle area, respectively.

$$\text{Muscle difference ratio}\left(\text{MDR}\right)=\frac{\begin{array}{l} The\;initial\;muscle\;mass\;area-\\ follow\;up\;muscle\;mass\;area \end{array}}{The\;initial\;muscle\;mass\;area}\times 100$$

### 2.4. Statistical analysis

All statistical analyses were performed using SPSS version 21 (SPSS Inc., Chicago, IL). Categorical variables are expressed as counts with percentages, whereas continuous variables are presented as the median with interquartile range. We divided the patients with COVID-19 into 2 groups: those with < 3 months of follow-up and those with ≥ 3 months of follow-up. The measurement of each muscle mass was performed by 2 independent readers. The interobserver variability for each chest wall mass was assessed using intraclass coefficient (ICC; 2-way random effects, absolute agreement). We compared the demographic characteristics, muscle index, and MDR of each muscle between the 2 groups using Mann–Whitney *U* test for continuous variables and chi-squared tests for categorical variables.

We investigated the relevant predictors of intercostal MDR ≥ 25% during the follow-up of COVID-19 using stepwise logistic regression analysis. For binary logistic regression, we used the mean value as the cutoff for the continuous dependent variable. We classified age < 65 years, female, ventilator use, steroid use, ≥3 months of follow-up duration, >13 days of hospital stay, and < 18.5 kg/m^2^ of body mass index as dependent variables. A *P* value of <.05 was considered statistically significant.

## 3. Results

### 3.1. Patient information

**Figure 2.**
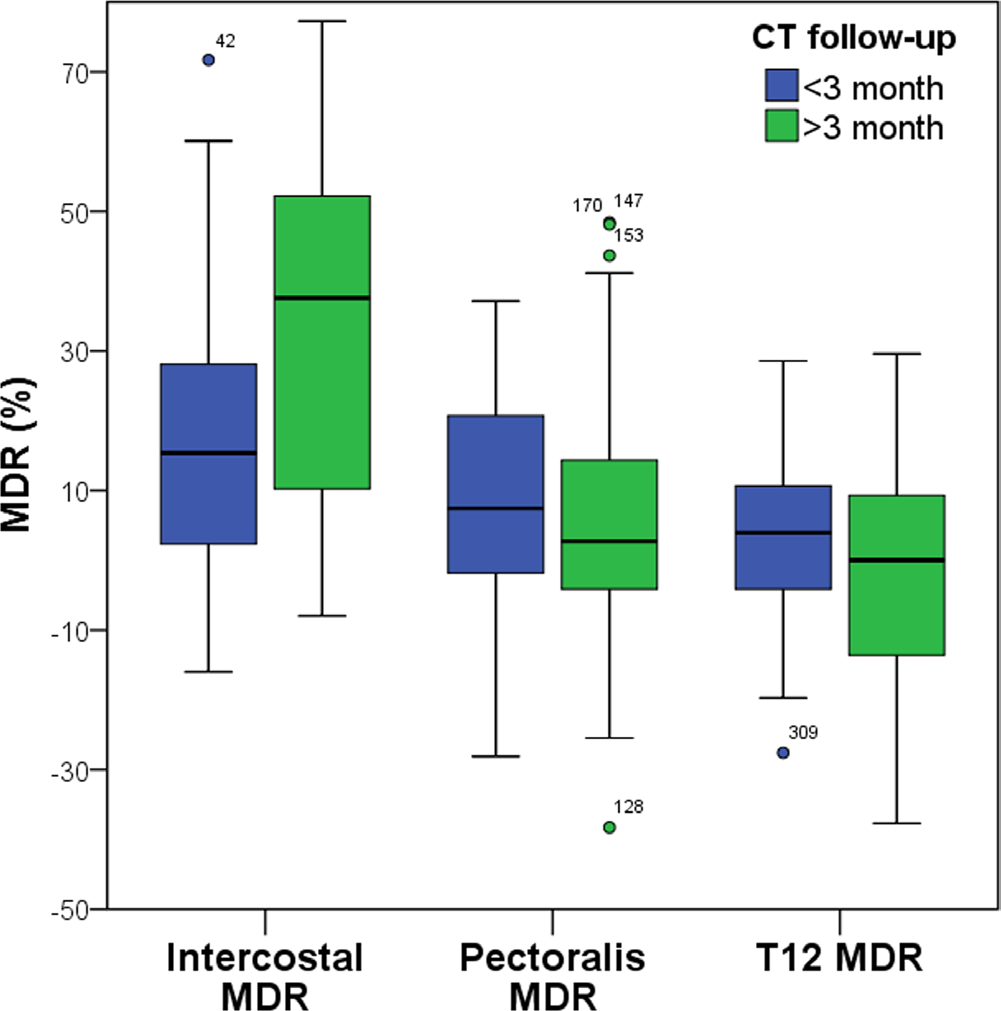
Comparison of intercostal MDR, pectoralis MDR, and T12 MDR between the groups with < 3 mo and ≥ 3 mo of follow-up. The intercostal MDR rate was significantly higher than the pectoralis and T12 MDR rates. The number of intercostal MDR bacteria was significantly higher in patients with ≥ 3 mo of follow-up than in those with < 3 mo of follow-up. MDR = muscle difference ratio, T12 = thoracic 12th vertebra level.

One hundred ten patients were included (37 females; median age: 68.5 [55.8–75.0] years old). The median period from the positive PCR test to the chest CT was 1.0 (0–7.3) days. The ICC was used to calculate the inter-reader reliability for each muscle mass measurement. The ICC values were ranged from 0.967 to 0.998, indicating excellent agreement (Table 1). We compared the degree of sarcopenia among the 3 chest wall compartments in the CT follow-up. A comparison of the demographics, muscle index and MDR of each muscle is shown in Table 2. The loss of intercostal muscle was the most severe, and T12 skeletal muscle was the least severe among the 3 chest wall muscles in the CT follow-up (Fig. 2). Intercostal MDR was significantly higher in the group with ≥ 3 months of follow-up than in the group with < 3 months of follow-up (32.5 ± 23.6% vs 19.0 ± 21.1%, *P* = .002). The pectoralis MDR (6.3 ± 20.3 vs 6.1 ± 19.0, *P* = .947) was not significantly different between the 2 groups. T12 MDR was slightly lower at ≥ 3 months of follow-up (−3.4 ± 18.8%) than at < 3 months of follow-up (2.7 ± 14.7%), but there was no significant difference between the 2 groups (*P* = .061). Each muscle index of the 3 muscle compartments was slightly lower in the group with ≥ 3 months of follow-up, but there were no significant differences between the 2 groups. Additionally, there were no significant differences between the 2 groups regarding underlying diseases, age, sex, body mass index, duration between the PCR test and CT scan, duration of hospitalization, mechanical ventilator and steroids.

**Table 1:** Interobserver agreement between the 2 readers in intercostal muscle mass, pectoralis muscle mass and T12 skeletal muscle mass.

Variables	Intraclass correlation coefficient (95% CI)
Intercostal muscle mass, initial	0.976 (0.948–0.989)
Pectoralis muscle mass, initial	0.970 (0.935–0.986)
T12 skeletal muscle mass, initial	0.998 (0.996–0.999)
Intercostal muscle mass, follow up	0.967 (0.931–0.984)
Pectoralis muscle mass, follow up	0.969 (0.935–0.985)
T12 skeletal muscle mass, follow up	0.990 (0.979–0.995)

T12 = thoracic 12th vertebra level.

**Table 2:** Comparison of demographics, clinical and CT findings in COVID-19 patients with < 3 mo of CT follow-up and those with ≥ 3 mo of CT follow-up.

	Follow-up < 3 mo (n = 53)	Follow-up > 3 mo (n = 57)	*P* value
Age (yr)	70.0 (55.0–80.0)	68.0 (56.0–73.0)	.305
Sex (male)	37 (69.8%)	36 (63.2%)	.461
Cardiovascular disease	11 (20.8%)	6 (10.5%)	.138
Hypertension	31 (58.5%)	30 (52.6%)	.537
Diabetes	22 (41.5%)	18 (31.6%)	.279
Chronic renal disease	4 (7.5%)	6 (10.5%)	.587
Cancer	7 (13.2%)	11 (19.3%)	.388
Duration between PCR test and CT scan	1.0 (0–7.0)	1.0 (0–8.0)	.760
CT Follow-up period (d)	31.0 (13.0–67.5)	165.0 (118.5–211.0)	<.001*
Hospital stay (d)	15.0 (9.5–29.0)	10.0 (7.5–22.5)	.054
Mechanical ventilation	6 (11.3%)	6 (10.5%)	.894
Steroid	36 (67.9%)	40 (70.2%)	.799
Body mass index	23.7 (21.4–26.3)	24.3 (20.7–26.7)	.546
Intercostal muscle index (cm^2^/m^2^)	95.5 (73.5–121.4)	93.9 (79.1–122.2)	.836
Pectoralis muscle index (cm^2^/m^2^)	1095.3 (904.7–1304.7)	1169.7 (900.9–1362.8)	.250
T12 muscle index (cm^2^/m^2^)	2545.3 (2049.3–2986.4)	2578.1 (2253.2–3256.1)	.174
Intercostal MDR (%)	15.4 (2.0–30.1)	37.6 (10.1–52.3)	.004*
Pectoralis MDR (%)	7.4 (−4.5 to 21.2)	2.7 (−4.4 to 16.8)	.482
T12 MDR (%)	4.0 (−4.8 to 10.9)	0.1 (−14.1 to 9.3)	.053

MDR = muscle difference ratio, PCR = polymerase chain reaction, T12 = thoracic vertebra at T12 level.

We investigated the contributing factors for intercostal MDR of ≥ 25% of patients with COVID-19. In the univariate regression analysis, steroid use and CT follow-up after 3 months were significant predictors for intercostal MDR ≥ 25%. After adjusting for confounding factors (age < 65 years, female, body mass index < 18.5 kg/m^2^, ventilator use, steroid use, follow-up > 3 months, hospital stay > 13 days), we found that steroid use (adjusted odds ratio (OR): 3.494, 95% confidence interval (CI): 1.419–8.604, *P* = .007) and CT follow-up after 3 months (adjusted OR: 3.006, 95% CI: 1.339–6.748, *P* = .008) were significant predictors of intercostal MDR ≥ 25% (R^2^ = 0.172, *P* = .006). These results are shown in Table 3.

**Table 3:** Predictors of intercostal muscle difference ratio ≥ 25% in follow-up of COVID-19 (R^2^ = 0.172, *P* = .006).

	Univariate regression analysis		Multivariate regression analysis	
	Adjusted odds ratio (95% confidence interval)	*P* value	Adjusted odds ratio (95% confidence interval)	*P* value
Steroid	3.300 (1.387–7.853)	.007*	3.494 (1.419–8.604)	.007
Follow-up ≥ 3 mo	2.847 (1.312–6.175)	.008*	3.006 (1.339–6.748)	.008
Age < 65 yr	2.097 (0.972–4.524)	.059		
Female	2.226 (0.990–5.006)	.053		
Body mass index < 18.5 kg/m^2^	4.077 (0.441–37.705)	.216		
Mechanical ventilation	1.042 (0.314–3.445)	.947		
Hospital stay > 13 d	1.077 (0.510–2.275)	.846		
Intercostal muscle index	1.933 (0.903–4.137)	.089		
Pectoralis muscle index	2.083 (0.974–4.457)	.059		
T12 muscle index	1.793 (0.841–3.824)	.131		

T12 = thoracic 12th vertebra level.

## 4. Discussion

The degree of chest wall mass wasting varied depending on their location in the follow-up CT of COVID-19. Among the 3 chest wall muscles, the loss of the intercostal muscle was the most severe, whereas the loss of T12 skeletal muscle was the least in the CT follow-up. In addition, the wasting of the intercostal muscle mass worsened, pectoralis muscle wasting persisted without additional deterioration, and T12 skeletal muscle mass minimally recovered during follow-up periods exceeding 3 months. These results support our speculation that both the intercostal and pectoralis muscles, serving as major and accessory respiratory muscles, may undergo varying degrees of injury following COVID-19 infection. Furthermore, the use of steroids and a follow-up period exceeding 3 months were identified as significant predictors of intercostal muscle wasting in follow-up scans.

The intercostal muscle suffered the most severe damage during the follow-up period, and this muscle wasting worsened after 3 months. Currently, there is no study that explores the association between intercostal muscle and infection. Among critically ill COVID-19 patients who required mechanical ventilation, approximately 40% to 50% patients exhibited pulmonary function abnormalities, such as reduced diffusion capacity in the 45 to 100 days of follow-up.^[14,15]^ While residual pulmonary fibrosis is the main cause of decreased diffusion capacity,^[15]^ we propose that the impairment of intercostal muscles might also be a potential contributing factor to pulmonary function abnormalities in survivors of COVID-19. The diaphragm, another crucial respiratory muscle, can be easily evaluated by ultrasonography. Previous research has identified diaphragmatic dysfunction by ultrasonography in patients with COVID-19 pneumonia who underwent mechanical ventilation.^[16,17]^ Additionally, one study reported that 7 of 9 patients demonstrated partial or full recovery after receiving proper respiratory physiotherapy in the 3- to 4-month follow-up of COVID-19.^[17]^ On the other hand, our patients did not undergo proper respiratory rehabilitation after COVID-19 infection, potentially leading to prolonged intercostal muscle wasting after 3 months of follow-up. These findings underscore the significance of implementing respiratory rehabilitation for COVID-19 survivors.

The T12 skeletal muscle mass decreased within 3 months of COVID-19 but minimally recovered after 3 months in our study. The skeletal muscle in T12 contains not only respiratory muscle, such as intercostal muscle but also nonrespiratory muscles, such as the external and internal oblique, erector spinae, rectus abdominis, and latissimus dorsi muscles. Consequently, we hypothesized that most nonrespiratory muscles tend to recover from muscular injuries after 3 months of COVID-19 infection. The significance of T12 skeletal muscle mass as a predictor in patients with COVID-19 has been controversial in prior research. A previous study reported that skeletal muscle mass at T12 was associated with the length of hospital stay in patients with COVID-19^.[4]^ Other studies demonstrated that skeletal muscle mass at T12 was not associated with 60- or 90-day mortality, admission to the intensive care unit, mechanical ventilation in patients with COVID-19,^[4,18]^ or 6-minute walking distance at the 3-month follow-up.^[19]^ These findings suggest that muscle wasting in the T12 region may not be a significant concern following COVID-19 infection. Another study found that fat accumulation within the erector spinae muscle in the initial CT contributed to the 6-minute walk distance after 3 months of COVID-19.^[19]^ This study underscores the importance of investigating the specific role of different muscles in acute infection, such as COVID-19.

We observed a reduction in pectoralis muscle mass during the follow-up period after COVID-19 infection. The pectoralis muscle mass was less decreased than the intercostal muscle mass but more decreased than the T12 skeletal muscle mass after COVID-19 infection. Since the pectoralis muscle plays a relatively minor role in respiration, it is possible that the muscular damage in the pectoralis muscle following COVID-19 may be less severe than that of the intercostal muscles. In previous studies, pectoralis muscle mass could be a prognostic factor for short-term mortality in COVID-19 patients,^[5,6,20]^ but those studies were limited to evaluations based on initial CT scans. Further research is necessary to assess the temporal changes in pectoralis muscle mass and its prognostic significance in long-term follow-up.

Steroids are commonly approved as a treatment option for critically ill COVID-19 patients.^[21–23]^ Our study revealed that steroid therapy is a significant factor in predicting the wasting of intercostal muscles during follow-up. Prior research has demonstrated that the use of steroids is associated with sarcopenia in patients with rheumatoid arthritis,^[24,25]^ chronic obstructive pulmonary disease, and interstitial lung disease.^[26]^ However, these studies primarily focused on assessing skeletal muscle function in the upper or lower extremities. Larger prospective studies are required to understand the potential harmful impact of steroid treatment on respiratory muscle dysfunction.

A previous study revealed that skeletal muscle wasting was associated with more challenging weaning processes and higher mortality rates in critically ill patients who underwent mechanical ventilation.^[27–29]^ Mechanical ventilation can lead to disuse atrophy and muscle weakness in the diaphragm.^[1]^ However, our study showed a different outcome: mechanical ventilation was not a predictor of intercostal muscle wasting during follow-up. The impact of mechanical ventilation on intercostal muscles remains uncertain, but we assumed that the relatively small number of patients who underwent mechanical ventilation might have influenced the results of our study.

This study had several limitations. First, it was conducted retrospectively in a single tertiary hospital, which may have induced selection bias. Some COVID-19 patients with severe pneumonia who required mechanical ventilation were transferred from other facilities, while a significant number of patients not in need of intensive care units were sent to secondary care hospitals due to bed shortages. Consequently, many patients were lost to follow-up. Second, we measured chest wall mass in both initial and follow-up CT scans, but the timing of these follow-up scans varied among patients. A prospective study with serial follow-up CT scans is necessary to obtain a better understanding of how muscular injury develops over time after COVID-19. Third, the intercostal muscle is a relatively small structure compared to the pectoralis muscle. Consequently, it is crucial that skilled radiologists outline the region of interest of the intercostal muscle accurately. Fourth, we were unable to determine the incidence of intercostal muscle wasting in patients with COVID-19 because there has been no investigation into the normal values of intercostal muscle mass. Fifth, because this study is retrospective, it was impossible to examine factors that may affect post-COVID-19 syndrome, such as systemic symptoms and muscle function decline. A large-scale prospective study is needed to evaluate the relationship between intercostal muscle wasting and other clinical factors in patients with post-COVID syndrome.

## 5. Conclusion

The intercostal muscle wasting was profound compared to that in the pectoralis muscle and T12 skeletal muscle in a follow-up CT scan. The use of steroids and a follow-up period exceeding 3 months were significant predictors for more than 25% of intercostal muscle wasting in follow-up. Rehabilitation of the intercostal muscles may be necessary to reduce the risk of long-COVID syndrome and pulmonary complications.

## Author contributions

**Conceptualization:** Mi Jung Park.

**Formal analysis:** Byeong Ju Koo, Hye Young Choi, Mi Jung Park.

**Investigation:** Ho Cheol Choi.

**Methodology:** Hwa Seon Shin, Mi Jung Park.

**Resources:** Jung Ho Won, Won Jeong Yang.

**Validation:** Seok Jin Hong, Jae Kyeong Ahn.

**Writing – original draft:** Byeong Ju Koo, Mi Jung Park.

**Writing – review & editing:** Mi Jung Park.
